# Supplementary material for: Intracellular Growth and Cell Cycle Progression are Dependent on (p)ppGpp Synthetase/Hydrolase in Brucella abortus
Source: Pathogens. 2020 Jul 14;9(7):571. doi: 10.3390/pathogens9070571 (PMC7400157; doi:10.3390/pathogens9070571)
Supplement: Supplementary file 1 [file pathogens-09-00571-s001.pdf]

Supplementary data

Content

Figure S1. Effect of *dksA* deletion on macrophage infection.
Figure S2. Codon-adapted *mesh1b* sequence
Figure S3. Fluorescence microscopy of the *pSRK-relA' mCherry-parB* strain induced with IPTG.
Table S1. Number of bacteria counted in Figure 5.

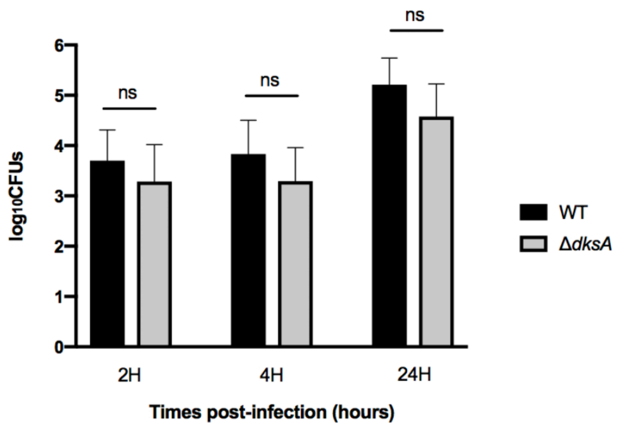

Figure S1. Survival of *B. abortus* WT and Δ*dksA* strains during infection of RAW 264.7 macrophages. Strains were grown in liquid culture overnight in order to reach exponential phase. Cultures were then diluted in DMEM to obtain a MOI of 50. Concentrations of live bacteria (log_{10}CFUs ml^{-1}) were determined at 0 h, 4 h, and 24 h post-infection by plating serial dilutions. Values represent the means of three independent experiments and the error bars represent the standard deviation. A student t test was performed as statistical analysis. The asterisks mean significant for $P < 0.05$ (*); $P < 0.01$ (**); $P < 0.0001$ (****).

ccccccgggatggccacctatccgtcggccaagttcatggaatgcctgcagtatgccgccttcaagcatcgccagcagcgccgcaag

gatccgcaggaaaccccgtatgtgaatcatgtgatcaatgtgtcgaccatcctgtcggtggaagcctgcatcaccgatgaaggcgtg

ctgatggccgccctgctgcatgatgtggtggaagataccgatgcctcgttcgaagatgtggaaaagctgttcggcccggatgtgtgcg

gcctggtgcgcgaagtgaccgatgataagtcgctggaaaagcaggaacgcaagcgcctgcagatcgaaaatgccgccaagtcgtc

gtgccgcgccaagctgatcaagctggccgataagctggataatctgcgcgatctgcaggtgaataccccgaccggctggacccagg

aacgccgcgatcagtatttcgtgtgggccaagaaggtggtggataatctgcgcggcaccaatgccaatctggaactgaagctggat

gaaatcttccgccagcgcggcctgctgtgaaagcttggg

Figure S2. Codon-adapted sequence of Mesh1b used in this study.

0H 2H

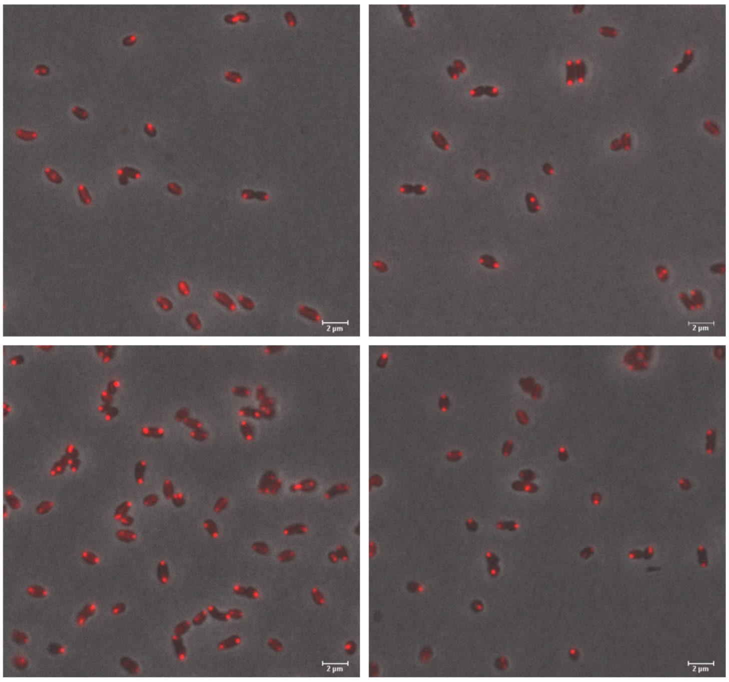

4H 6H

Figure S3. Fluorescence microscopy of the *pSRK-relA' mCherry-parB* strain induced with IPTG. Bacteria were grown in liquid culture (2YT medium) overnight in order to reach exponential phase. Culture was then diluted to an OD of 0.1 in 2YT medium supplemented with IPTG. Samples were taken every 2 hours, placed on a PBS agarose pad and observed with a fluorescence microscope. Bacteria in G1 phase (presenting only one focus of mCherry-ParB in red) were counted for each time post-induction. Scale bars represent 2 μm.

Time	pSRK-*relA'*	pSRK-*relA'* +IPTG	pSRK-*relA'**	pSRK-*relA'** +IPTG
**0 h**	262	262	289	289
	341	341	356	356
	291	291	307	291
**2 h**	260	286	268	260
	283	300	321	297
	302	317	287	285
**4 h**	243	275	246	236
	312	393	291	295
	282	411	317	344
**6 h**	285	326	252	257
	317	355	296	288
	327	431	311	341

Table S1. Number of bacteria considered (n) in the G1 counting experiment (Figure 5) for each strain with or without IPTG and at each time post-induction.
